# Weighing in on the Heavy Psychological Tolls of Obesity∗

**DOI:** 10.1016/j.jacadv.2024.101114

**Published:** 2024-07-23

**Authors:** Carl J. Lavie, Deepika R. Laddu, Ross Arena

**Affiliations:** a Department of Cardiovascular Diseases, John Ochsner Heart and Vascular Institute, Ochsner Clinical School-The University of Queensland School of Medicine, New Orleans, Louisiana, USA; b Healthy Living Pandemic Event Protection (HL-PIVOT) Network, Chicago, Illinois, USA; c Arbor Research, Ann Arbor, Michigan, USA; d Department of Physical Therapy, College of Applied Science, University of Illinois Chicago, Chicago, Illinois, USA

**Keywords:** fitness, obesity, psychological stress, mental health, physical activity

**Figure:**
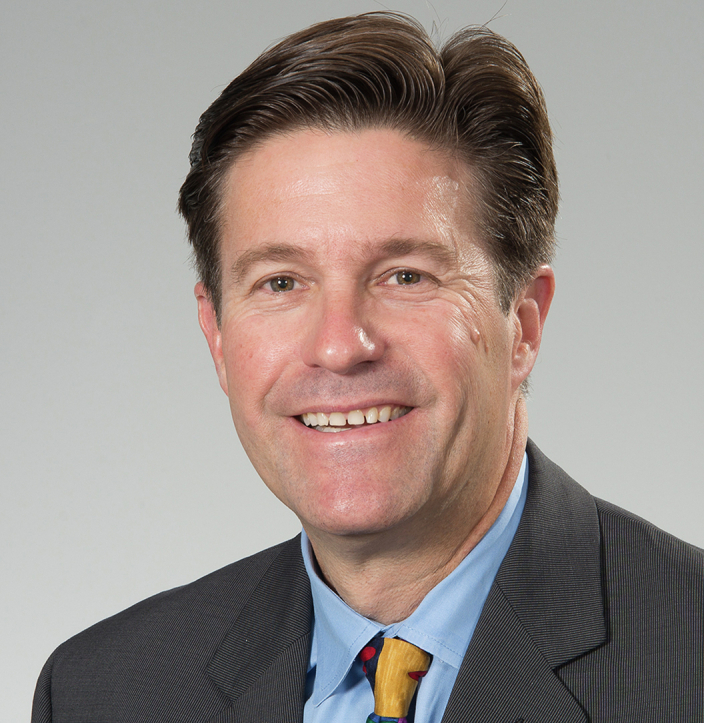

In the United States, obesity is increasing at epidemic levels in adults and children/adolescents and markedly increases the prevalence of cardiometabolic abnormalities, which are major precursors to cardiovascular diseases (CVDs).^1^, ^2^, ^3^ Obesity is also associated with an increased risk of psychological distress (PD) and has adverse effects on mental health (MH).^4^^,^^5^ Both obesity and PD have worsened considerably during the COVID-19 pandemic.^6^^,^^7^ Since PD, including depression, anxiety, hostility/unexpressed anger, are associated with a greater risk of CVD and a worse prognosis for CVD,^8^ this combination certainly places a great threat on the overall health of society and, especially, increases the long-term risk of CVD.

In this issue of *JACC: Advances*, Kundi et al^9^ presented data from the 2013 to 2018 (pre-COVID-19) National Health Interview Survey in 20,954 young individuals aged 18 to 26 years on body mass index (BMI) and PD. In this young population, already 27% were overweight, 24% were obese, and 5.1% had severe Class III obesity (BMI ≥40 kg/m^2^). The PD was adjusted for: 1) age alone; 2) age, sex, and race; 3) age, sex, race, and comorbidities; 4) age, sex, race, and social determinants of health (SDoH); including economic stability, neighborhood/physical environment, community and social context, food insecurity, education, and health care system; and 5) fully adjusted by age, sex, race, comorbidities, and SDoH. In the unadjusted model, all obesity classes were associated with PD. Following adjustment for the above factors, the association remained significant for the higher obesity classes (Class II [BMI 35-39.9 kg/m^2^] and III [BMI ≥40 kg/m^2^]). Importantly, those individuals with Class III obesity were 40% more likely to have PD. In subgroup analyses, the results were similar in non-Hispanic Whites and females but not in Hispanics, non-Hispanic Blacks, and male subgroups.

In this study, it was determined that heightened levels of PD are positively associated with obesity and that this relationship is the strongest for young adults with Class II and Class III obesity. On top of sociocultural factors influencing the relationship between PD and obesity, the biological connection between PD and obesity is additionally well-characterized. Obesity can lead to dysfunction of the hypothalamic-pituitary-adrenal axis, resulting in elevated cortisol levels and feelings of PD.^3^

There are long-term implications and bi-directional links between obesity and MH.^10^ The consequences of childhood and adolescent obesity extend beyond PD, and obesity has been shown to precede severe behavioral and MH disorders. Depression, anxiety, eating disorders, attention-deficit/hyperactivity disorder, and poor psychosocial well-being are more prevalent in children and adolescents with obesity than in healthy-weight youth.^11^ Clearly, public health messages need to consider the overlay of PD in relation to obesity; MH messaging should be woven into weight loss messaging in an appropriate way.^12^

Structured behavioral-based lifestyle modification interventions demonstrate promise in addressing and preventing both physiological and psychological comorbidities while promoting overall well-being in youth with obesity,^13^ similar to what we have recently reported with lifestyle interventions in adults with obesity.^14^^,^^15^ Data from a systematic review and meta-analysis examining the impact of lifestyle-based obesity treatment interventions on depression and anxiety symptoms in children and adolescents with overweight or obesity showed that structured and professionally managed obesity treatments that include a dietary component and/or physical activity (PA) are associated with short-term improvements in depression and anxiety, despite there being no differences in weight-related outcomes.^13^ Similarly, the same authors reported that structured obesity treatment interventions with a dietary component have been shown to reduce the prevalence and risk of eating disorders, as well as related symptoms, in children and adolescents with overweight or obesity, both in the short term and up to 6 years from baseline.^16^ Clearly, meeting the PA recommendations is crucial for achieving good health with the benefits extending to PD and MH in children and adults.^3^^,^^13^^,^^17^ We have demonstrated in adults that PA/exercise interventions reduce PD, especially depression, and lower stress-induced mortality risk, as we have reviewed elsewhere.^3^^,^^17^

We have now moved into the era of pharmacological intervention in obesity, with very effective agents that work via glucagon-like peptide 1 receptor agonists and/or gastric inhibitory polypeptide mechanisms to induce 15% to 25% weight reductions, which have not been seen previously with nonpharmacological/lifestyle interventions and are now rivaling the weight loss with bariatric/metabolic surgeries. However, in this pharmacologic era, the interaction between MH and weight loss needs to be considered. The current body of evidence is mixed: some studies show a mix of marked improvements, some demonstrate marked deteriorations in mood,^18^ whereas other studies suggest improvements in MH.^19^^,^^20^ Obviously, longer-term studies are needed, with and without PA/exercise training, to assess impacts on PD, MH, and short- and long-term CVD prognosis.

Finally, the authors of this study should be applauded for highlighting the impact of obesity, especially in moderate and severe forms, on PD, even in fairly young adults. Integrating screening for PD or MH disorders into routine obesity treatment protocols could help identify PD early, allowing for timely intervention and support. As we have discussed recently in other *JACC* publications, since atherosclerosis and CVD begin early in life, efforts at early prevention would be ideal.^21^, ^22^, ^23^, ^24^ Certainly efforts to prevent obesity and PD early in life, particularly through healthy lifestyle interventions that have been shown to have beneficial effects on both, such as diet or nutrition education, PA, exercise training, and fitness improvements,^3^^,^^13^^,^^17^ would be excellent starting points for preventive cardiology.

## Funding support and author disclosures

Dr Lavie serves on the DSMB for Novo Nordisk. All other authors have reported that they have no relationships relevant to the contents of this paper to disclose.

## References

[1] Liu J., Zhang Y., Lavie C.J., Moran A.E. (2022). Trends in metabolic phenotypes according to body mass index among US Adults, 1999-2018. Mayo Clin Proc.

[2] Liu J., Mark P.Y.M., Ma J., Lavie C.J. (2023). Trends in metabolic phenotypes of obesity among US adolescents, NHANES 1999-2018. Mayo Clin Proc.

[3] Lavie C.J., Laddu D., Arena R., Ortega F.B., Alpert M.A., Kushner R.F. (2018). Healthy weight and obesity prevention: JACC health promotion series. J Am Coll Cardiol.

[4] Steptoe A., Frank P. (2023). Obesity and psychological distress. Philos Trans R Soc B Biol Sci.

[5] Sarwer D.B., Polonsky H.M. (2016). The psychosocial burden of obesity. Endocrinol Metab Clin North Am.

[6] Arena R., Hall G., Laddu D.R., Phillips S.A., Lavie C.J. (2022). A tale of two pandemics revisited: physical inactivity, sedentary behavior and poor COVID-19 outcomes reside in the same Syndemic City. Prog Cardiovasc Dis.

[7] Dubey S., Biswas P., Ghosh R. (2020). Psychosocial impact of COVID-19. Diabetes Metab Syndr.

[8] Lavie C.J., Menezes A.R., De Schutter A., Milani R.V., Blumenthal J.A. (2016). Impact of cardiac rehabilitation and exercise training on psychological risk factors and subsequent prognosis in patients with cardiovascular disease. Can J Cardiol.

[9] Kundi H., Rahman Z.M., Friedman M. (2024). Association of obesity with psychological distress in young adults: patterns by gender and race/ethnicity. JACC: Adv.

[10] Leutner M., Dervic E., Bellach L., Klimek P., Thurner S., Kautzky A. (2023). Obesity as pleiotropic risk state for metabolic and mental health throughout life. Transl Psychiatry.

[11] Galler A., Thönnes A., Joas J. (2024). Clinical characteristics and outcomes of children, adolescents and young adults with overweight or obesity and mental health disorders. Int J Obes.

[12] Van Buren D.J., Sinton M.M. (2009). Psychological aspects of weight loss and weight maintenance. J Am Diet Assoc.

[13] Jebeile H., Gow M.L., Baur L.A., Garnett S.P., Paxton S.J., Lister N.B. (2019). Association of pediatric obesity treatment, including a dietary component, with change in depression and anxiety: a systematic review and meta-analysis. JAMA Pediatr.

[14] Katzmarzyk P.T., Martin C.K., Newton R.L. (2020). Weight loss in underserved patients - a cluster-randomized trial. N Engl J Med.

[15] Höchsmann C., Dorling J.L., Martin C.K. (2021). Effects of a 2-year primary care lifestyle intervention on cardiometabolic risk factors: a cluster-randomized trial. Circulation.

[16] Jebeile H., Gow M.L., Baur L.A., Garnett S.P., Paxton S.J., Lister N.B. (2019). Treatment of obesity, with a dietary component, and eating disorder risk in children and adolescents: a systematic review with meta-analysis. Obes Rev.

[17] Popovic D., Bjelobrk M., Tesic M. (2022). Defining the importance of stress reduction in managing cardiovascular disease - the role of exercise. Prog Cardiovasc Dis.

[18] Arillotta D., Floresta G., Guirguis A. (2023). GLP-1 Receptor agonists and related mental health issues; insights from a range of social media platforms using a mixed-methods approach. Brain Sci.

[19] Chen X., Zhao P., Wang W., Guo L., Pan Q. (2024). The antidepressant effects of GLP-1 receptor agonists: a systematic review and meta-analysis. Am J Geriatr Psychiatry.

[20] Kim Y.K., Kim O.Y., Song J. (2020). Alleviation of depression by glucagon-like peptide 1 through the regulation of neuroinflammation, neurotransmitters, neurogenesis, and synaptic function. Front Pharmacol.

[21] Fuster V. (2023). Paradigm Shift: children present the most critical point of engagement for choosing cardiovascular health. J Am Coll Cardiol.

[22] Ventura H.O., Elagizi A., Lavie C.J. (2023). Optimal prevention of cardiovascular diseases: the earlier the better. J Am Coll Cardiol.

[23] Santos-Beneit G., Bodega P., Cos-Gandoy de (2024). Effect of time-varying exposure to school-based health promotion on adiposity in childhood. J Am Coll Cardiol.

[24] Lavie C.J., Neeland I.J., Ortega F.B. (2024). Intervention in school age children to prevent progression of obesity and cardiometabolic disease-a paradigm shift indeed. J Am Coll Cardiol.

